# Complete mitochondrial genome of the spotted lanternfly, *Lycorma delicatula* White, 1845 (Hemiptera: Fulgoridae)

**DOI:** 10.1080/23802359.2019.1703577

**Published:** 2020-01-16

**Authors:** Na Ra Jeong, Min Jee Kim, Wonhoon Lee, Gwan-Seok Lee, Iksoo Kim

**Affiliations:** a Department of Applied Biology, College of Agriculture and Life Sciences, Chonnam National University, Gwangju, Republic of Korea;; b Herbal Medicine Resources Research Center, Korea Institute of Oriental Medicine, Naju, Republic of Korea;; c Department of Plant Medicine and Institute of Agriculture and Life Sciences, Gyeongsang National University, Jinju, Republic of Korea;; d Department of Agro-food Safety and Crop Protection, Crop Protection Division, National Institute of Agricultural Sciences, RDA, Wanju, Republic of Korea

**Keywords:** *Lycorma delicatula*, mitochondrial genome, Fulgoridae

## Abstract

The spotted lanternfly, *Lycorma delicatula* White, 1845 (Hemiptera: Fulgoridae), is an invasive pest that attacks forest as well as agricultural trees. We sequenced the 15,798-bp long complete mitochondrial genome (mitogenome) of this species; it consists of a typical set of genes (13 protein-coding genes, 2 rRNA genes, and 22 tRNA genes) and one major non-coding A + T-rich region. The orientation and gene order of the *L. delicatula* mitogenome are identical to that of the ancestral type found in majority of the insects. Bayesian inference (BI) and maximum-likelihood (ML) phylogeny placed the *L. delicatula* examined in our study, together with other geographical samples of the species in a group with the highest nodal support, forming the subfamily Aphaeninae to which *L. delicatula* belongs.

## Introduction

The spotted lanternfly, *Lycorma delicatula* White, 1845 (Hemiptera: Fulgoridae), is native to northern China (Liu 1939) and was detected as an exotic species in South Korea in 2004 (Kim and Kim 2005) and in Pennsylvania, USA in 2015 (Barringer et al. 2015). This pest damages a wide variety of forest trees, and particularly the agricultural grape tree, by feeding on the phloem sap and secreting honeydew, thereby inhibiting transpiration and leading to growth of sooty mold on the trees (Han et al. 2008; Lee et al. 2009; Park et al. 2009; Dara et al. 2015).

In previous studies, mitochondrial NADH dehydrogenase (ND) subunit 2 (ND2) and ND6 regions were analyzed from specimens collected from China, Korea, and Japan (Kim et al. 2013). However, specimens from Korea and Japan revealed identical sequences, warranting the need for variable markers for population genetics data.

## Methods

For our study, one wild adult lanternfly was caught on the tree of heaven (*Ailanthus altissima*) in the Nam-gu, Gwangju Metropolitan City, Republic of Korea (35°05′07.2′′ N, 126°52′02.0′′ E) and its DNA was extracted from one of the hind legs. Leftover DNA and the specimen were deposited at the Chonnam National University, Gwangju, Korea, under the accession number CNU11113.

Using the extracted DNA, four long overlapping fragments (LFs: *COI*-*trnN*, *COIII*-*CytB*, *ND6*-*srRNA*, and *lrRNA*-*COI*) were amplified using four sets of primers designed using data regarding the previously published species of Fulgoroidea, with special consideration for geographically close specimens of *L. delicatula*, published in earlier studies (Hua et al. 2009; Song et al. 2012). Using the LFs as templates, 36 overlapping short fragments (SF) were amplified using the aforementioned primers.

**Figure 1.**
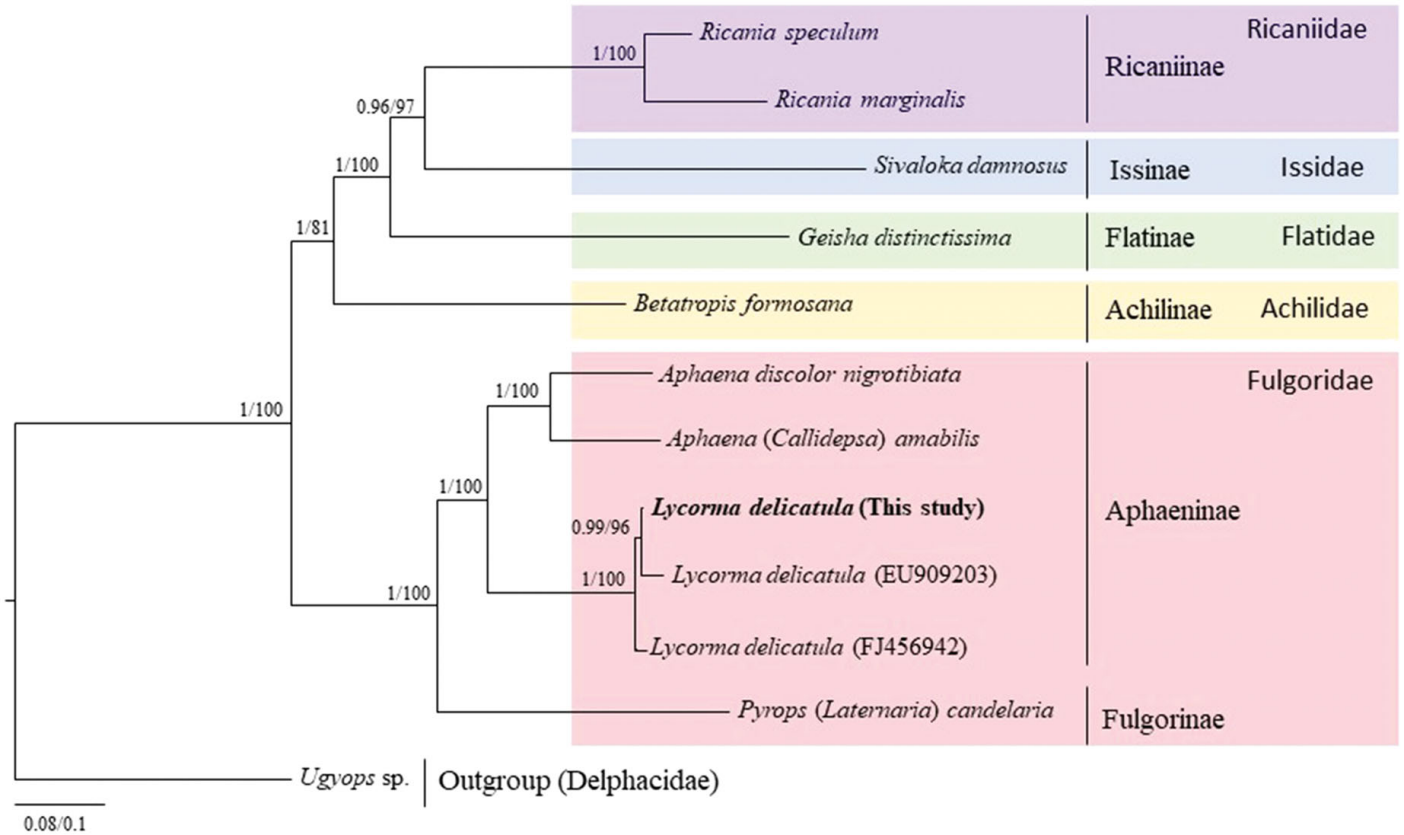
Phylogenetic tree for the superfamily Fulgoroidea. The tree was constructed using the concatenated 13 PCGs and 2 rRNAs *via* the maximum-likelihood (ML) and Bayesian Inference (BI) methods. The numbers at each node specify bootstrap percentages of 1000 pseudoreplicates by ML analysis and Bayesian posterior probabilities in percent by BI analysis. The scale bar indicates the number of substitutions per site. Delphacidae (*Ugyops* sp., MH352481, Yu and Liang 2018) was used as outgroup. GenBank accession numbers are as follows: *Ricania speculum*, KX371891 (Zhang et al. 2016); *Ricania marginalis*, JN242415 Song et al. 2012); *Sivaloka damnosus*, FJ360694 (Song et al. 2010); *Geisha distinctissima*, FJ230961 (Song and Liang 2009); *Betatropis formosana*, MH324927 (Xu et al. 2019); *Aphaena discolor nigrotibiata*, MN025523 (Wang et al. 2019); *Aphaena (Callidepsa) amabilis*, MN025522 (Wang et al. 2019); *Lycorma delicatula*, EU909203 (Song et al. 2012); *Lycorma delicatula*, FJ456942 (Hua et al. 2009); and *Pyrops (Laternaria) candelaria*, FJ006724 (Song et al. 2012).

Phylogenetic analysis was performed using 11 available mitogenomes from Fulgoroidea, including the one obtained in this study (Figure 1). Nucleotide sequences of all protein-coding genes (PCGs) and rRNAs were aligned and well-aligned blocks were selected using GBlocks 0.91b software (Castresana 2000) with the maximum number of contiguous non-conserved positions set to 11 and no gap positions allowed. Subsequently, 13 PCGs and 2 rRNAs were concatenated in alignment (11,301 bp excluding gaps). Bayesian inference (BI) and maximum-likelihood (ML) methods were applied using MrBayes version 3.2.6 (Ronquist et al. 2012) and RAxML-HPC2 version 8.0.24 (Stamatakis 2014), respectively, which were incorporated into the CIPRES Portal version 3.1 (Miller et al. 2010). An optimal partitioning scheme (nine partitions) and substitution model (GTR + Gamma + I) were determined using PartitionFinder 2 with the Greedy algorithm (Lanfear et al. 2012, 2014, 2016). Phylogenetic trees were visualized using FigTree version 1.42 (http://tree.bio.ed.ac.uk/software/figtree/).

## Results

The *L. delicatula* mitogenome was found to be 15,789 bp in length, with typical gene sets – 2 rRNAs, 22 tRNAs, and 13 PCGs – and a major non-coding A + T-rich region of 1495 bp length (GenBank accession number MN607209), whereas previous studies showed that the mitogenome was 15,946 bp long (Song et al. 2012) and 15,410 bp (Hua et al. 2009). The largest size variation was detected in the A + T-rich region (1043 bp in Hua et al. (2009), 1495 bp in this study, and 1642 bp in Song et al. (2012)). The gene arrangement of *L. delicatula* was identical to that of the ancestral type found in majority of the insects (Boore 1999).

Phylogenetic analyses using both, BI and ML methods, using 13 PCGs and two rRNAs, placed *L. delicatula* from Korea, along with previously analyzed geographical samples, into one group, with the highest nodal support in both analyses. The subfamily Aphaeninae, to which *L. delicatula* belongs, forms a cohesive monophyletic group with the highest nodal supports indicated by BI and ML analyses.
